# Strategic Sampling of Eurasian Otter Spraints for Genetic Research in South Korea: Enhancing PCR Success and Data Accuracy

**DOI:** 10.3390/ani15040574

**Published:** 2025-02-17

**Authors:** Jee Hyun Kim, Jangmi Lee, Dong Youn Kim, Yoon-Do Yang, Sujoo Cho, Han-Chan Park, Sung Yong Han, Mi-Sook Min, Hang Lee, Je-Yoel Cho, Puneet Pandey

**Affiliations:** 1 Research Institute for Veterinary Science, College of Veterinary Medicine, Seoul National University, Seoul 08826, Republic of Korea; kjhsonic@hanmail.net (J.H.K.); jmhr15@naver.com (J.L.); dreamcat2017@gmail.com (D.Y.K.); rhkcjs6450@naver.com (S.C.); minbio@yahoo.co.kr (M.-S.M.); hanglee@snu.ac.kr (H.L.); 2 Conservation Genome Resource Bank for Korean Wildlife, Seoul 08826, Republic of Korea; hanchanee@gmail.com; 3 Veterinary Humanities and Social Science, College of Veterinary Medicine, Seoul National University, Seoul 08826, Republic of Korea; 4 Department of Biological Sciences, Konkuk University, Seoul 05029, Republic of Korea; rkddkwldbseh@naver.com; 5 Department of Agriculture, Forestry and Bioresources, College of Agriculture and Life Sciences, Seoul National University, Seoul 08826, Republic of Korea; 6 Association of Korean Otter Conservation, Hwacheon 24135, Republic of Korea; hsy5034@hanmail.net; 7 Department of Biochemistry, College of Veterinary Medicine, Research Institute for Veterinary Science, BK21 FOUR Future Veterinary Medicine Leading Education and Research Center, Seoul National University, Seoul 08826, Republic of Korea; 8 Comparative Medicine Disease Research Center (CDRC), Seoul National University, Seoul 08826, Republic of Korea

**Keywords:** non-invasive sampling, Eurasian otter, DNA degradation, PCR amplification success, genotyping errors

## Abstract

Non-invasive genetic approaches, particularly using fecal samples, are commonly used to study endangered and elusive species, as they are easy to collect with minimal permission and cause little disturbance to the subject population. However, such studies face limitations due to poor DNA yield, which affects the overall utilization of collected samples and increases data errors. Here, we evaluated the impact of sample age and collection season on the performance of DNA extracted from feces (spraints) of the Eurasian otter (*Lutra lutra*), a semi-aquatic apex predator in South Korean freshwater ecosystems. We found that PCR amplification success rates decreased more rapidly in summer (79.3–58.2%) compared to winter (99.2–84.8%) with extended environmental exposure. Genotyping error rates were higher in samples collected during summer, with the rate of error increase over time being significantly greater in summer than in winter. The hot and humid South Korean summer fosters microbial growth and fecal degradation, which negatively impacts DNA yield, reducing PCR amplification success and increasing genotyping errors. We recommend collecting otter feces during winter for better DNA quality. If sampling in summer is unavoidable, it is crucial to collect fresh samples, which can be facilitated by conducting frequent surveys of latrine sites.

## Simple Summary

Non-invasive genetic approaches using fecal samples are valuable for studying endangered species, but they face challenges like poor DNA yield and increased data errors. This study evaluated the impact of sample age and season on DNA extracted from Eurasian otter spraints (feces) in South Korea. We found that PCR amplification success rates and genotyping accuracy decreased more rapidly in summer due to higher microbial growth and fecal degradation. Winter samples showed better DNA quality. We recommend collecting otter spraints in winter or, if summer sampling is necessary, using fresh samples from frequently monitored latrine sites.

## 1. Introduction

Understanding genetic diversity, its maintenance, and enrichment, as well as leveraging this knowledge to develop species management and restoration plans for endangered species, is essential [1,2]. Such efforts ensure the species’ evolutionary potential to adapt to changing environments, and maintain reproductive success [3,4]. The overall success and reliability of genetic studies depend on the quantity and quality of DNA obtained from collected biological samples, which ensure high marker amplification efficiency and minimize the risk of data errors. However, securing high-quality biological material, such as blood or tissue, from endangered species is particularly challenging due to their small population sizes and high conservation protection. Wildlife capture to collect samples can negatively affect their health, and even if samples are obtained from captured or deceased individuals, the total number is often insufficient to accurately determine the genetic status of a population [5,6,7,8]. Therefore, genetic studies using non-invasive samples have gained popularity over recent decades for investigating endangered and elusive species [9,10,11,12,13]. Fecal samples, in particular, are favored because their collection is cost-effective, and a larger sample size can be easily acquired within shorter study durations compared to invasive and other non-invasive sampling methods [14,15,16]. However, fecal samples often yield low molecular weight, degraded DNA that may contain environmental contaminants, prey DNA, and inhibitory compounds, which can reduce PCR amplification success rates and increase the likelihood of errors in the generated data [17,18,19,20,21,22]. Additionally, climatic factors such as temperature, humidity, and the duration of environmental exposure significantly affect DNA yield from fecal samples [23,24,25,26,27,28,29].

The Eurasian otter (*Lutra lutra*) is a semi-aquatic carnivore belonging to the mustelid family [30]. The species is a key apex predator in freshwater ecosystems in South Korea. Genetic management of this species is essential, as it has been designated an endangered species in South Korea due to historical population declines and habitat degradation [31,32]. Given their elusive behavior, genetic research on otters has predominantly relied on non-invasive sampling methods [33,34,35,36,37,38,39]. Despite this, the impact of essential factors in non-invasive sampling—such as sample age, sampling season, DNA extraction protocols, and PCR strategies—on the reliability and success of genetic studies remains inadequately explored, with no comprehensive assessments conducted in Korea to date [40,41,42].

The characteristics of defecation sites (e.g., rocks on riverbeds) and the Korean climate (humid summers and dry winters) necessitate efforts to optimize strategies for otter feces (spraints) collection, including the timing (time between defecation and sample collection) and season of sampling. However, such efforts have yet to be undertaken. Otters deposit spraint with high moisture content on rocks near riverbeds, making it prone to degradation if not collected promptly. In winter, spraints are prone to rapid desiccation under extremely low temperatures and humidity, which can complicate sample homogenization during DNA extraction and negatively impact DNA yield. Conversely, in summer, high temperatures, humidity, and frequent precipitation accelerate microbial activity and DNA degradation, while heavy rainfall often washes spraints away before collection.

This study aims to optimize fecal collection strategies to enhance the reliability of genetic analysis and research accuracy. We conducted experiments by exposing otter spraints to outdoor conditions under natural experiment conditions, assessing fecal DNA quality through PCR amplification success rates and genotyping error rates (allelic dropout and false alleles).

## 2. Materials and Methods

This research was conducted in compliance with legal and ethical regulations, and the protocol was approved by the Seoul National University Institutional Animal Care and Use Committee. All sample collections were performed with the utmost effort to minimize animal suffering.

**Figure 1:**
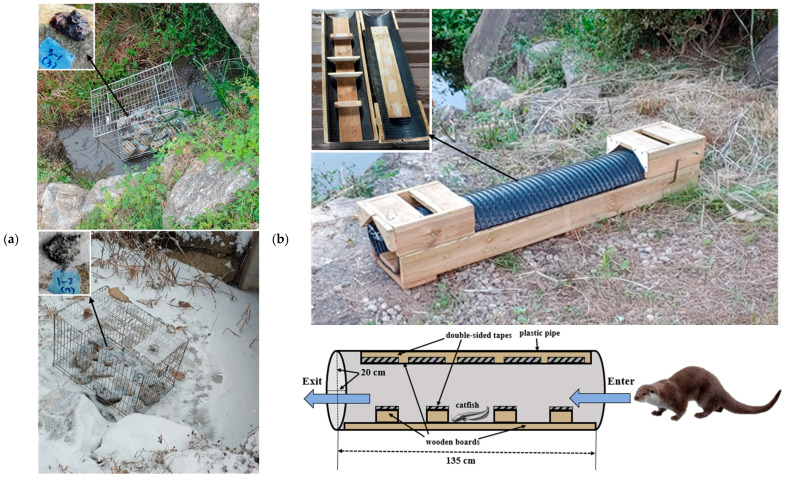
(**a**) Metal enclosure with otter spraints exposed to semi-natural conditions in summer (**upper**) and winter (**below**). (**b**) Hair trap used to collect otter hairs.

Otter spraints were collected from captive Eurasian otters (*L. lutra*) housed at the Korea Otter Research Center in Hwacheon, Gangwon-do, Republic of Korea. Sample collections were conducted in two distinct seasons—summer (August–September 2021) and winter (January–February 2022). Prior to sampling, old spraints were completely removed, and fresh spraints were collected within 4–5 h of defecation. Each collected otter spraint was divided into five equal fragments, with one fragment immediately preserved in absolute ethanol and referred to as day-zero (day 0) sample. The remaining four fragments were then placed on rocks within metal enclosures (90 cm width × 55 cm length × 65 cm height; Figure 1a) positioned over water (which freezes in winter) to expose them to natural conditions for 1, 3, 5 and 7 days, respectively. A temperature and humidity logger recorded and saved data every 15 min, close to the spraints. After the designated exposure periods, the samples were retrieved, placed in a plastic tube containing absolute ethanol, and stored at −70 °C until analysis. We collected otter spraints from each individual multiple times. Additionally, hair samples (with roots) were collected from each otter using a hair trap (Figure 1b), and the genotypes obtained from these samples served as the reference or true genotype. The hair trap was constructed from plastic pipe with wooden boards inside, and double-sided tape was affixed to hold bait (catfish) placed at the center [43]. Hairs were naturally collected by the tape as otters approached the bait.

DNA extraction from otter spraints was performed using the Gentra Puregene Tissue kit (QIAGEN, Hilden, Germany) with a modified protocol. A 1.6 mL aliquot of fecal suspension in absolute ethanol was transferred into a 2 mL tube and centrifuged to pellet the debris. After removing the ethanol, the fecal substrate (70–100 mg) was retained. We added 800 μL of ASL buffer (QIAGEN, Hilden, Germany) to the substrate, and after centrifugation, 600 μL of the supernatant was transferred to a 1.5 mL tube. A 100 μL aliquot of this supernatant was then lysed with 500 μL of cell lysis solution buffer and 3 μL of Proteinase K (QIAGEN, Hilden, Germany) at 55 °C for 3 h. The remaining steps followed the standard protocol.

DNA from hairs of each otter was extracted from at least 10 strands by cutting approximately 0.5 cm of the hair roots and incubating them in 150 μL of 10% Chelex-100 (Bio-Rad, Hercules, CA, USA) with 1.5 μL of Proteinase K (Thermo Scientific, Vilnius, Lithuania) at 55 °C overnight. The samples were then incubated at 100 °C for 20 min and centrifuged for 5 min. The supernatant was transferred to a new 1.5 mL tube and stored at −20 °C until use.

A total of 10 microsatellite markers were amplified in four multiplex PCR sets: Set 1—Lut453, 717; Set 2—Lut 604, 615, 715; Set 3—Lut 435, 832, 833; Set 4—Lut 457, 902 [44,45]. PCR amplifications were conducted in a 10 μL reaction volume consisting of 5 μL Multiplex PCR Master, 2 μL Q-solution (QIAGEN, Hilden, Germany), 1 μL Primer mix (2 μM each of primers), 1 μL BSA, and 1 μL of template DNA. Cycling conditions were as follows: initial denaturation at 95 °C for 15 min; 15 cycles of 94 °C for 30 s, 60 °C (−0.8 °C per cycle) for 90 s, and 72 °C for 1 min; followed by 25 cycles of 94 °C for 30 s, 48 °C for 90 s, and 72 °C for 1 min, with a final extension at 60 °C for 30 min.

Genotyping for hair DNA was performed in triplicate following the multi-tube approach, with additional PCRs conducted up to seven times, if needed. A genotype was deemed reliable if the allele pattern appeared at least five times for homozygous and at least two times for heterozygous genotypes. For spraint DNA, genotyping was conducted in phases. Initially, Set 1 (Lut453, 717) was amplified following the multi-tube approach (4 replicates) for DNA from all collected spraints (day 0 samples). Samples were considered to have positive amplification if at least 75% (three out of four) of the PCR attempts were successful, as determined by 1.5% agarose gel electrophoresis. The day 0, 1, 3, 5, and 7 samples from these screened spraints were then subjected to microsatellite genotyping in the same manner as the hair DNA. Fragment analysis was conducted using an ABI3730XL Genetic Analyzer (Applied Biosystems, Foster City, CA, USA) and allele scoring was performed with GeneMapper v 3.7 (Applied Biosystems, Foster City, CA, USA).

PCR amplification success rate (PCR+) was calculated as the proportion of successful amplifications to total PCRs [46]. Allelic dropout (ADO) was measured as the ratio of cases in which only one allele was amplified in heterozygous genotypes, while false alleles (FA) were identified as erroneous genotypes among the amplified genotypes [47]. Genotyping errors were assessed by comparing the results with genotypes obtained from hair samples.

A repeated measures two-way ANOVA was used to examine the effects of season, time, and their interaction on three response variables (PCR amplification success rate, allelic dropout, and false alleles). To utilize non-parametric data in the ANOVA, an Aligned Rank Transform was applied using the R package ‘ARTool’ [48,49]. Spearman’s correlation analysis was performed to examine the relationship between weather conditions (average temperature and relative humidity) and response variables. We used the average temperatures and relative humidity during the fecal exposure period as weather data. All statistical analyses were conducted using R software v4.4.1.

## 3. Results

### 3.1. Subject Otters and Sample Screening

A total of 62 fresh spraints were collected from captive individuals. During the summer survey, five sampling events were conducted, resulting in 28 spraints from six different otters. Of these, 22 samples underwent initial screening (Set 1), with 20 samples (mean weight: 2.41 ± 1.03 g) meeting the selection criteria and included in the experiment. In winter, 34 spraint samples were collected from three otters over four sampling events. After screening, 26 samples were assessed, and 20 samples (mean weight: 2.05 ± 1.05 g) were ultimately used in the experiments.

### 3.2. Weather Conditions

In summer, temperatures near the spraints ranged from 13.4 °C to 41.4 °C, with an average of 21.8 °C, while relative humidity varied between 35.5% and 97.9%, averaging 76.6% (Figure S1a). In winter, temperatures fluctuated between −14.4 °C and 13.0 °C, with an average of −4.0 °C, and relative humidity ranged from 21.1% to 86.9%, averaging 60.4% (Figure S1b).

### 3.3. PCR Amplification Success

**Figure 2:**
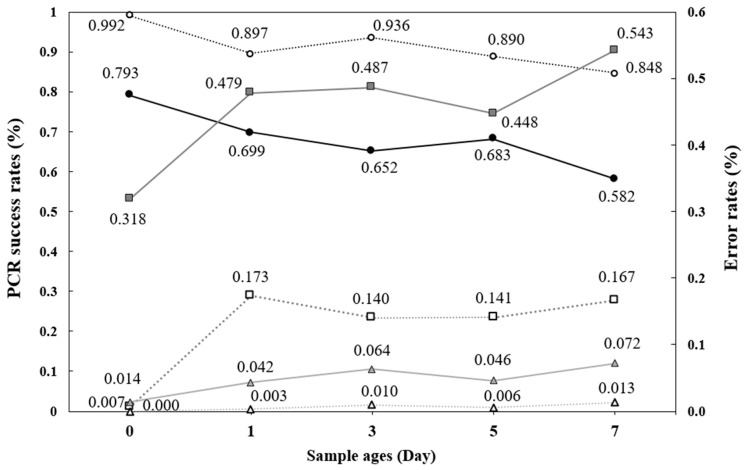
Average PCR success rates from DNA extracted from spraints collected in summer (●) and winter (○), along with the average of allelic dropout rates [summer (■) and winter (□)] and false allele rates [summer (▲) and winter (△)] over time. PCR success and error rates are presented on a scale from 0 to 1, where 0 indicates no success or no errors, and 1 represents 100% success or 100% error.

The average PCR amplification success rate (hereafter PCR success rate) of otter spraint DNA was consistently higher in winter than in summer across all sample ages (Figure 2), with a significant difference between the two seasons (Table 1; *p* < 0.001). The variance between samples was also greater in summer than in winter (Figure S2a). In both seasons, PCR success rates tended to decrease over time, with the highest rates observed on day 0, minor fluctuations between days 1 and 5, and the lowest rates on day 7 (Figure 2). Exposure time significantly affected PCR success rates (Table 1; *p* < 0.001). There was an interaction effect between season and time, with the amplification rate decreasing more rapidly over time in summer compared to winter (Table 1; *p* = 0.0019). Additionally, a moderate negative correlation was found between PCR success rate and both temperature and humidity (Table S1), suggesting that increases in these factors accelerate DNA degradation, thereby reducing PCR success.

### 3.4. Genotyping Errors

Allelic dropout (ADO) and false allele (FA) rates were higher in summer than in winter (Figure 2). A repeated measures two-way ANOVA confirmed significant seasonal differences in these rates (Table 1; *p* < 0.001). Summer samples exhibited greater variability compared to winter samples (Figure S2b,c). Although ADO and FA rates showed slight fluctuations from days 1 to 5, they remained higher across all sample ages compared to the freshest samples, with time having a significant impact on error rates (Table 1; *p* < 0.001). There were significant interaction effects between season and time for ADO and FA (Table 1; *p* < 0.05 and *p* < 0.001, respectively). Both ADO and FA rates were positively correlated with average temperature and humidity (Table S1), suggesting that higher temperatures and humidity levels contribute to elevated genotyping errors.

## 4. Discussion

This study evaluated the influence of seasonal environmental variables (temperature and humidity) and sample exposure duration on otter spraints to improve sample collection strategies. We analyzed PCR success and genotyping error rates across 10 microsatellite loci, finding higher PCR success rates in winter than in summer, with success decreasing over time in both seasons (Figure 2). Similarly, genotyping error rates were lower in winter and increased with extended environmental exposure (Figure 2). Variability was also greater in summer samples compared to those collected in winter across all response variables, which was significantly linked to average temperature and humidity (Figure S2, Table S1).

The accelerated degradation of otter spraint DNA in summer likely results from increased nuclease activity driven by higher temperatures and humidity. DNases, enzymes that cleave phosphodiester bonds in DNA, lead to DNA fragmentation and degradation [50,51], and their activity is heightened in warm, humid conditions [52]. Elevated temperatures also promote microbial growth within the spraints, accelerating decomposition and DNA degradation [53]. Consequently, spraints containing initially low quantities of otter DNA underwent further degradation, leading to reduced PCR success rates and increased genotyping errors.

In contrast, winter’s lower temperatures and humidity appear to slow fecal DNA degradation, maintaining relatively high PCR success and low error rates up to seven days post-deposition. Winter at the study site had an average temperature of −4.0 °C (ranging from −14.4 °C to 13.0 °C), consistently remaining below freezing except during daytime (10:00–16:00). The site was noticeably cold, and the low temperatures and snow cover likely preserved the frozen state of epithelial cells within the spraints [27]. This frozen state resembles the effects of rapid freezing shortly after defecation, slowing decomposition. However, daytime temperatures occasionally rose above freezing, causing snowmelt and subsequent DNA degradation. This seasonal effect aligns with prior studies, which observed that higher temperatures and humidity accelerate DNA degradation in spraints [27,28,54].

The allele dropout (ADO) and false allele (FA) rates were higher in summer and appeared more rapidly over time (Figure 2, Table 1). High genotyping error rates are common with non- invasive genetic samples like feces or spraints due to DNA’s typically poor quality and low quantity [55]. Therefore, adopting a multitube approach with repeated trials is recommended to achieve accurate results with these samples [20].

The fecal DNA degraded rapidly during a short time (day 1), leading to an increase in the average error rate, particularly ADO (Figure 2). However, between days 1 and 5, the average error rates did not show a gradual change in both seasons (Figure 2). This might be observed due to the short time intervals, which caused inconsistent outcomes among samples (Figure S2). In other words, while there were variations for samples, the short time intervals made it appear as though there were no noticeable differences over time on average. If the time intervals had been set wider, these detailed fluctuations might not have been detected. For example, Carpenter and Dziminski [56] studied bilby fecal DNA degradation with sampling intervals of 1, 7, 14, 21, 30, 90, and 180 days, and found little differences in PCR success or error rates between 1 and 7 days.

We expected that DNA degradation in otter spraints would become pronounced over extended exposure times, even in winter, as reported by Lampa, Gruber, Henle and Hoehn [40], who assessed the impact of storage time on DNA quality. They found 80% PCR success for DNA extracted on day 1, followed by a decrease to 63% and 60% for samples stored for one and two weeks at −20 °C. However, both studies employed different sample storage methodologies. Lampa, Gruber, Henle and Hoehn [40] sampled otter spraints using cotton swabs, while in our study, an intact portion of the spraint was sampled, exposed, stored in ethanol, and processed for DNA extraction. Swab samples typically contain fewer targeted otter epithelial cells, and these cotton swabs pose challenges for sample homogenization and the separation of epithelial cells during DNA extraction [57]. In contrast, this study utilized fecal suspensions preserved directly in ethanol, enabling easier epithelial cell separation and a more efficient DNA extraction process.

## 5. Conclusions

Studies aimed at understanding the genetic vigor of species require systematic and representative population sampling, which is often unfeasible through invasive or disruptive genetic sampling methods, particularly for rare and elusive species. However, while non-invasive sampling is considered effective, it has limitations, including low success rates that result in the loss of many samples, sometimes entirely missing the genetic contribution of certain individuals [58,59]. Additionally, it is prone to increased genetic errors, which can lead to an overestimation of genetic diversity and an underestimation of inbreeding levels compared to the actual population dynamics [60,61].

Genetic research using non-invasive samples has seen remarkable progress over recent decades, powered by advances in DNA extraction protocols and statistical methods that address errors and biases. For rare, elusive, and endangered species, obtaining biological samples can be challenging, even with non-invasive techniques. To overcome these limitations and maximize the efficient use of available samples, optimizing sampling strategies is essential.

In our study, a higher PCR success rate and fewer genotyping errors were observed in otter spraints collected during the winter compared to those collected in the summer, suggesting that winter is a more optimal season for otter spraint sampling in South Korea. If summer collection is necessary, weather conditions should be considered, avoiding days with high temperatures and humidity. Additionally, frequent visits to latrine sites are advised to collect fresh spraints within hours of deposition, maximizing DNA quality.

This study, conducted on captive otters, controlled for dietary variables that could affect DNA degradation rates. However, wild otters consume a varied diet, which may influence fecal DNA degradation differently. In future studies, extending the time intervals and maximum exposure duration would be valuable to pinpoint when fecal DNA degradation becomes significant in winter conditions.

## Figures and Tables

**Table 1:** Results of repeated-measures two-way ANOVA comparing response variables (PCR success and genotyping error rates) between seasons at the same sample age.

Response Variables	Sources	d.f.	d.f. Residual	F	*p*-Value
PCR success	Season	1	38	34.6873	8.0686 × 10^−7^ ***
	Time	4	152	15.9841	6.0601 × 10^−11^ ***
	Season × Time	4	152	4.4676	0.0019 **
Allelic dropout	Season	1	38	34.0171	9.6663 × 10^−7^ ***
	Time	4	152	11.2986	4.7136 × 10^−8^ ***
	Season × Time	4	152	2.7925	0.0283 *
False allele	Season	1	38	47.5331	3.4182 × 10^−8^ ***
	Time	4	152	13.9084	1.0844 × 10^−9^ ***
	Season × Time	4	152	6.1242	0.0001 ***

‘*’: *p* < 0.05, ‘**’: *p* < 0.01, and ‘***’: *p* < 0.001.

## Data Availability

The data that support the findings of this study are included within the article and its Supplementary Materials file. The raw datasets used for analysis are available from the corresponding author upon reasonable request.

## Supplementary Materials

The following supporting information can be downloaded at: https://www.mdpi.com/article/10.3390/ani15040574/s1, Figure S1. Graphs of daily weather data during the survey period: temperature (°C) and relative humidity (%). (**a**) summer: black arrows mark the timing of heavy rain (**b**) winter: black arrows indicate the time points when snow fell. Figure S2: Boxplots showing the distribution of (**a**) PCR success, (**b**) allelic dropout, and (**c**) false allele rates from spraints collected over time in both summer and winter (*n* = 20 per sample age). The median values are indicated by the lines within each box, and black dots represent outliers in the data. Table S1: Correlation analysis between response variables (PCR success, allelic dropout, and false allele rates) and weather data (average temperature and relative humidity) in summer and winter. Values presented in the table are Spearman’s correlation coefficients (rho), which indicate the strength and direction of the relationship between variables. The values in parentheses are *p*-values, which are used to determine significance.

## References

[1] Weeks A.R., Heinze D., Perrin L., Stoklosa J., Hoffmann A.A., van Rooyen A., Kelly T., Mansergh I. (2017). Genetic rescue increases fitness and aids rapid recovery of an endangered marsupial population. Nat. Commun..

[2] Poirier M.A., Coltman D.W., Pelletier F., Jorgenson J., Festa-Bianchet M. (2019). Genetic decline, restoration and rescue of an isolated ungulate population. Evol. Appl..

[3] Allendorf F.W., Luikart G.H., Aitken S.N. (2012). Conservation and the Genetics of Populations.

[4] Frankham R., Briscoe D.A., Ballou J.D. (2002). Introduction to Conservation Genetics.

[5] Wasser S., Houston C., Koehler G.M., Cadd G., Fain S. (1997). Techniques for application of faecal DNA methods to field studies of Ursids. Mol. Ecol..

[6] Pauli J.N., Whiteman J.P., Riley M.D., Middleton A.D. (2010). Defining noninvasive approaches for sampling of vertebrates. Conserv. Biol..

[7] Harcourt R.G., Turner E., Hall A., Waas J.R., Hindell M. (2010). Effects of capture stress on free-ranging, reproductively active male Weddell seals. J. Comp. Physiol. A.

[8] Huber N., Vetter S.G., Evans A.L., Kjellander P., Küker S., Bergvall U.A., Arnemo J.M. (2017). Quantifying capture stress in free ranging European roe deer (*Capreolus capreolus*). BMC Vet. Res..

[9] Andrews K.R., De Barba M., Russello M.A., Waits L.P. (2021). Advances in using non-invasive, archival, and environmental samples for population genomic studies. Population Genomics: Wildlife.

[10] Zemanova M.A. (2021). Noninvasive genetic assessment is an effective wildlife research tool when compared with other approaches. Genes.

[11] Cho S., Pandey P., Hyun J.Y., Marchenkova T., Vitkalova A., Petrov T., Jeong D., Lee J., Kim D.Y., Li Y. (2022). Efficient and cost-effective non-invasive population monitoring as a method to assess the genetic diversity of the last remaining population of Amur leopard (*Panthera pardus orientalis*) in the Russia Far East. PLoS ONE.

[12] Jeong D., Hyun J.Y., Marchenkova T., Matiukhina D., Cho S., Lee J., Kim D.Y., Li Y., Darman Y., Min M.-S. (2024). Genetic Insights and Conservation Strategies for Amur Tigers in Southwest Primorye, Russia. Sci. Rep..

[13] Li Y., Kim J.H., Li H., Peng Y., Chen M., Zhu W., Pandey P., Sedash G., Wang T., Darman Y. (2022). Northward range expansion of water deer in Northeast Asia: Direct evidence and management implications. Animals.

[14] Ferreira C.M., Sabino-Marques H., Barbosa S., Costa P., Encarnação C., Alpizar-Jara R., Pita R., Beja P., Mira A., Searle J.B. (2018). Genetic non-invasive sampling (gNIS) as a cost-effective tool for monitoring elusive small mammals. Eur. J. Wildl. Res..

[15] Singh S.K., Mishra S., Aspi J., Kvist L., Nigam P., Pandey P., Sharma R., Goyal S.P. (2015). Tigers of Sundarbans in India: Is the population a separate conservation unit?. PLoS ONE.

[16] Singh S.K., Aspi J., Kvist L., Sharma R., Pandey P., Mishra S., Singh R., Agrawal M., Goyal S.P. (2017). Fine-scale population genetic structure of the Bengal tiger (*Panthera tigris* tigris) in a human-dominated western Terai Arc Landscape, India. PLoS ONE.

[17] Taberlet P., Luikart G. (1999). Non-invasive genetic sampling and individual identification. Biol. J. Linn. Soc..

[18] Fabbri E., Caniglia R., Mucci N., Thomsen H.P., Krag K., Pertoldi C., Loeschcke V., Randi E. (2012). Comparison of single nucleotide polymorphisms and microsatellites in non-invasive genetic monitoring of a wolf population. Arch. Biol. Sci..

[19] Kohn M.H., Wayne R.K. (1997). Facts from feces revisited. Trends Ecol. Evol..

[20] Taberlet P., Griffin S., Goossens B., Questiau S., Manceau V., Escaravage N., Waits L.P., Bouvet J. (1996). Reliable genotyping of samples with very low DNA quantities using PCR. Nucleic Acids Res..

[21] Sittenthaler M., Schöll E.M., Leeb C., Haring E., Parz-Gollner R., Hackländer K. (2021). Factors influencing genotyping success and genotyping error rate of Eurasian otter (*Lutra lutra*) faeces collected in temperate Central Europe. Eur. J. Wildl. Res..

[22] Taberlet P., Waits L.P., Luikart G. (1999). Noninvasive genetic sampling: Look before you leap. Trends Ecol. Evol..

[23] Murphy M.A., Kendall K.C., Robinson A., Waits L.P. (2007). The impact of time and field conditions on brown bear (*Ursus arctos*) faecal DNA amplification. Conserv. Genet..

[24] Brinkman T.J., Schwartz M.K., Person D.K., Pilgrim K.L., Hundertmark K.J. (2010). Effects of time and rainfall on PCR success using DNA extracted from deer fecal pellets. Conserv. Genet..

[25] DeMay S.M., Becker P.A., Eidson C.A., Rachlow J.L., Johnson T.R., Waits L.P. (2013). Evaluating DNA degradation rates in faecal pellets of the endangered pygmy rabbit. Mol. Ecol. Resour..

[26] Lonsinger R.C., Gese E.M., Dempsey S.J., Kluever B.M., Johnson T.R., Waits L.P. (2015). Balancing sample accumulation and DNA degradation rates to optimize noninvasive genetic sampling of sympatric carnivores. Mol. Ecol. Resour..

[27] Lucchini V., Fabbri E., Marucco F., Ricci S., Boitani L., Randi E. (2002). Noninvasive molecular tracking of colonizing wolf (*Canis lupus*) packs in the western Italian Alps. Mol. Ecol..

[28] Piggott M.P. (2004). Effect of sample age and season of collection on the reliability of microsatellite genotyping of faecal DNA. Wildl. Res..

[29] King S.R., Schoenecker K.A., Fike J.A., Oyler-McCance S.J. (2018). Long-term persistence of horse fecal DNA in the environment makes equids particularly good candidates for noninvasive sampling. Ecol. Evol..

[30] Basnet A., Ghimire P., Timilsina Y.P., Bist B.S. (2020). Otter research in Asia: Trends, biases and future directions. Glob. Ecol. Conserv..

[31] Han S.Y. (1997). The Ecological Studies of Eurasian Otter (Lutra lutra) in South Korea.

[32] National Institute of Ecology Ministry of Environment. https://www.nie.re.kr/nieEng/main/contents.do?menuNo=400052.

[33] Balestrieri A., Gariano P., Grandinetti M., Verduci F., Gianfranceschi L., Gatti E., Mucci N., Mengoni C., Tremolada P. (2022). Faecal DNA-based genetic survey of a relict Eurasian otter (*Lutra lutra*) population (Sila Massif, S Italy). Conserv. Genet. Resour..

[34] Prigioni C., Remonti L., Balestrieri A., Sgrosso S., Priore G., Mucci N., Randi E. (2006). Estimation of European otter (*Lutra lutra*) population size by fecal DNA typing in southern Italy. J. Mammal..

[35] Vergara M., Ruiz-González A., de Luzuriaga J.L., Gómez-Moliner B.J. (2014). Individual identification and distribution assessment of otters (*Lutra lutra*) through non-invasive genetic sampling: Recovery of an endangered species in the Basque Country (Northern Spain). Mamm. Biol..

[36] Park J., Min M.S., Jeong D.C., Han S.W., Kim J.H., Cha H.G., Park H.C. (2022). Identification of individuals and kinship using Eurasian otter fecal DNA from the Naeseongcheon stream. J. Asia-Pac. Biodivers..

[37] Kim H.-J., Han S.-Y., Sasaki T., Ogawa H., Yamamoto K., Ando M. (2023). Distribution of the Eurasian otter (*Lutra lutra*) on isolated islands of Korea. J. Environ. Inf. Sci..

[38] Park C.-S., Cho G.-J. (2017). Individual identification of Eurasian otters (*Lutra lutra*) in South Korea (Sincheon River, Daegu) by microsatellite markers. J. Vet. Med. Sci..

[39] Hájková P., Zemanová B., Roche K., Hájek B. (2009). An evaluation of field and noninvasive genetic methods for estimating Eurasian otter population size. Conserv. Genet..

[40] Lampa S., Gruber B., Henle K., Hoehn M. (2008). An optimisation approach to increase DNA amplification success of otter faeces. Conserv. Genet..

[41] Hájková P., Zemanová B., Bryja J., Hájek B., Roche K., Tkadlec E., Zima J. (2006). Factors affecting success of PCR amplification of microsatellite loci from otter faeces. Mol. Ecol. Notes.

[42] Lerone L., Mengoni C., Carpaneto G.M., Randi E., Loy A. (2014). Procedures to genotype problematic non-invasive otter (*Lutra lutra*) samples. Acta Theriol..

[43] Kuhn R.A. (2010). Note on Hair-Sampling Devices for Eurasian Otters. IUCN Otter Spec. Group Bull..

[44] Dallas J., Piertney S. (1998). Microsatellite primers for the Eurasian otter. Mol. Ecol..

[45] Dallas J.F., Bacon P.J., Carss D.N., Conroy J.W., Green R., Jefferies D.J., Kruuk H., Marshall F., Piertney S.B., Racey P.A. (1999). Genetic diversity in the Eurasian otter, *Lutra lutra*, in Scotland. Evidence from microsatellite polymorphism. Biol. J. Linn. Soc..

[46] Santini A., Lucchini V., Fabbri E., Randi E. (2007). Ageing and environmental factors affect PCR success in wolf (*Canis lupus*) excremental DNA samples. Mol. Ecol. Notes.

[47] Broquet T., Petit E. (2004). Quantifying genotyping errors in noninvasive population genetics. Mol. Ecol..

[48] Kay M., Elkin L.A., Higgins J.J., Wobbrock J.O. ARTool: Aligned Rank Transform for Nonparametric Factorial ANOVAs. R Package Version 0.11.1. https://github.com/mjskay/ARTool.

[49] Wobbrock J.O., Findlater L., Gergle D., Higgins J.J. The aligned rank transform for nonparametric factorial analyses using only anova procedures. Proceedings of the SIGCHI Conference on Human Factors in Computing Systems.

[50] Matsuo Y., Yamada A., Tsukamoto K., Tamura H.O., Ikezawa H., Nakamura H., Nishikawa K. (1996). A distant evolutionary relationship between bacterial sphingomyelinase and mammalian DNase I. Protein Sci..

[51] Laskowski M. (1967). DNases and their use in the studies of primary structure of nucleic acids. Adv. Enzymol. Relat. Areas Mol. Biol..

[52] Liao C., Zhang M., Cheng X., Li Q., Mao F., Wang X., Yu C., Yu Z., Jia Y., Li J. (2020). Identification and characterization of the nuclease activity of the extracellular proteins from Salmonella enterica serovar Typhimurium. Curr. Microbiol..

[53] Zhang M., Wei M., Dong Z., Duan H., Mao S., Feng S., Li W., Sun Z., Li J., Yan K. (2019). Fecal DNA isolation and degradation in clam Cyclina sinensis: Noninvasive DNA isolation for conservation and genetic assessment. BMC Biotechnol..

[54] Rea R.V., Johnson C.J., Murray B.W., Hodder D.P., Crowley S.M. (2016). Timing moose pellet collections to increase genotyping success of fecal DNA. J. Fish Wildl. Manag..

[55] Morin P.A., Chambers K.E., Boesch C., Vigilant L. (2001). Quantitative polymerase chain reaction analysis of DNA from noninvasive samples for accurate microsatellite genotyping of wild chimpanzees (*Pan troglodytes verus*). Mol. Ecol..

[56] Carpenter F.M., Dziminski M.A. (2016). Breaking down scats: Degradation of DNA from greater bilby (*Macrotis lagotis*) faecal pellets. Aust. Mammal..

[57] Miles K.A., Holtz M.N., Lounsberry Z.T., Sacks B.N. (2015). A paired comparison of scat-collecting versus scat-swabbing methods for noninvasive recovery of mesocarnivore DNA from an arid environment. Wildl. Soc. Bull..

[58] Adams J.R., Waits L.P. (2007). An efficient method for screening faecal DNA genotypes and detecting new individuals and hybrids in the red wolf (*Canis rufus*) experimental population area. Conserv. Genet..

[59] Kohn M.H., York E.C., Kamradt D.A., Haught G., Sauvajot R.M., Wayne R.K. (1999). Estimating population size by genotyping faeces. Proc. R. Soc. Lond. Ser. B Biol. Sci..

[60] Schultz A.J., Strickland K., Cristescu R.H., Hanger J., de Villiers D., Frère C.H. (2022). Testing the effectiveness of genetic monitoring using genetic non-invasive sampling. Ecol. Evol..

[61] Creel S., Spong G., Sands J.L., Rotella J., Zeigle J., Joe L., Murphy K.M., Smith D. (2003). Population size estimation in Yellowstone wolves with error-prone noninvasive microsatellite genotypes. Mol. Ecol..

