# Supplementary material for: Strategic Sampling of Eurasian Otter Spraints for Genetic Research in South Korea: Enhancing PCR Success and Data Accuracy
Source: Animals (Basel). 2025 Feb 17;15(4):574. doi: 10.3390/ani15040574 (PMC11851711; doi:10.3390/ani15040574)
Supplement: Supplementary file 1 [file animals-15-00574-s001.zip › animals-3429967-supplementary.pdf]

Supplementary Materials

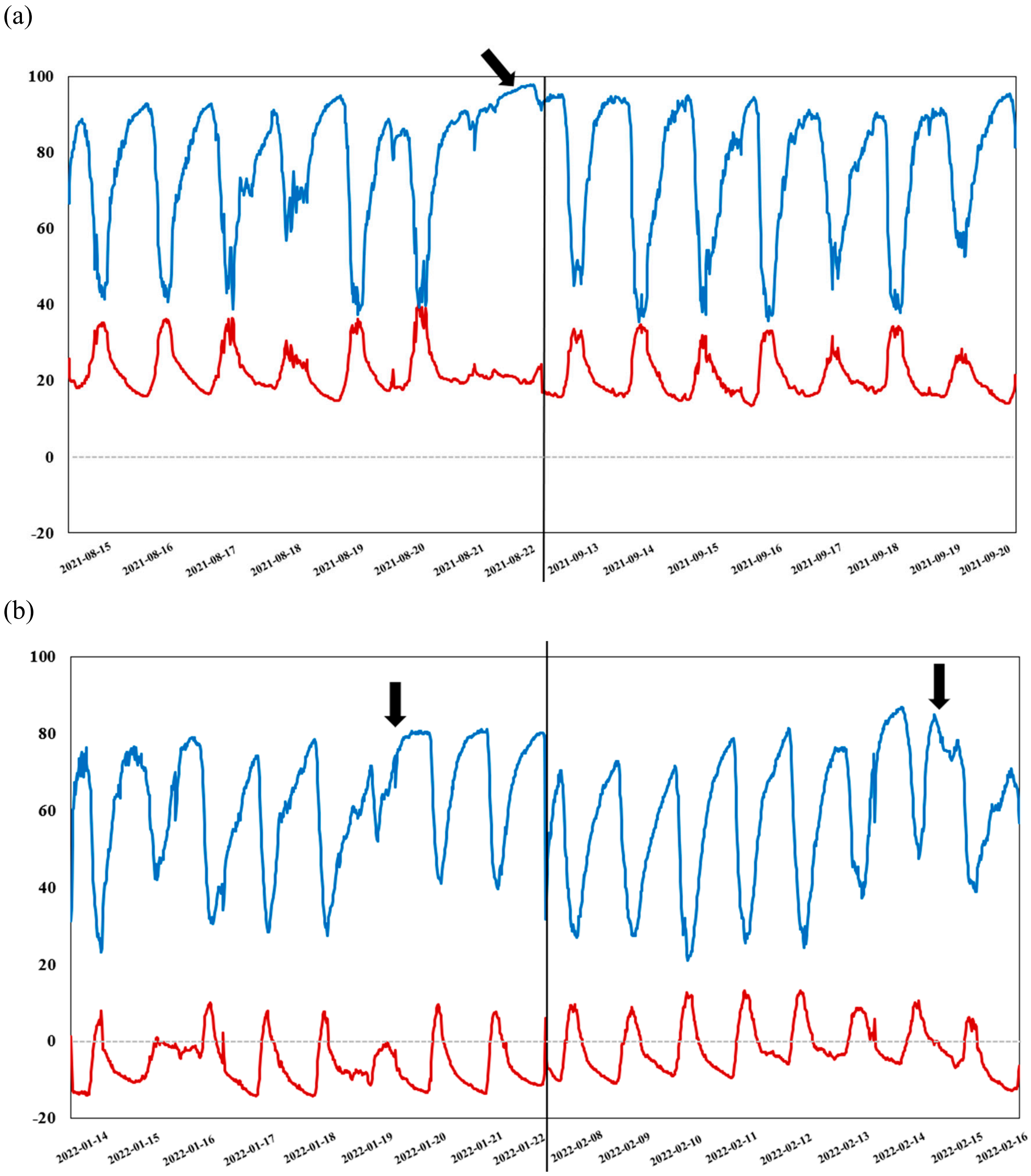

Figure S1. Graphs of daily weather data during the survey period: temperature (℃) and relative humidity (%). (a) summer: black arrows mark the timing of heavy rain (b) winter: black arrows indicate the time points when snow fell.

(a)

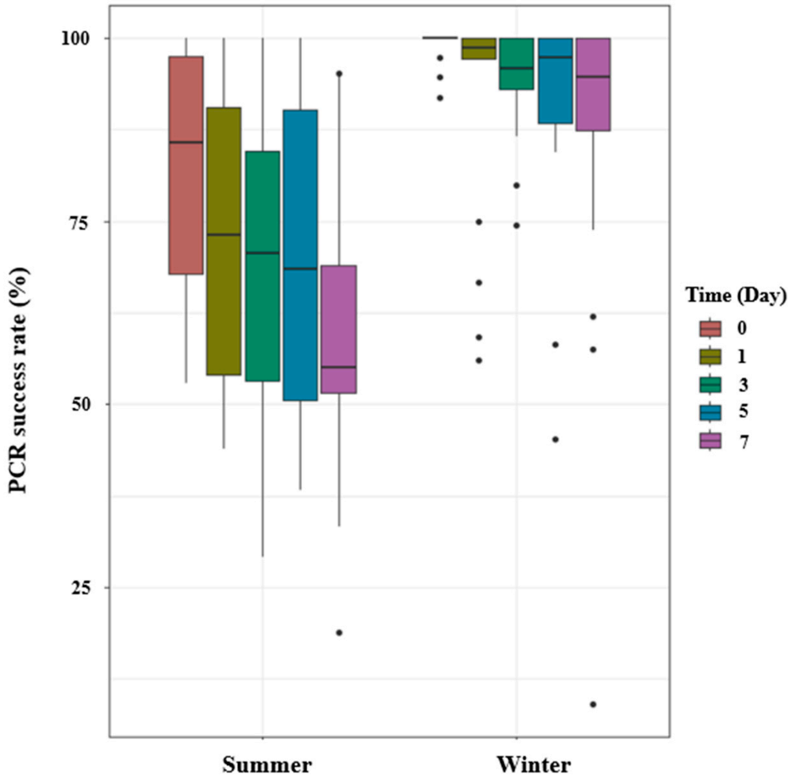
100
75
50
25
PCR success rate (%)
Summer
Winter
Time (Day)
0
1
3
5
7

(b)

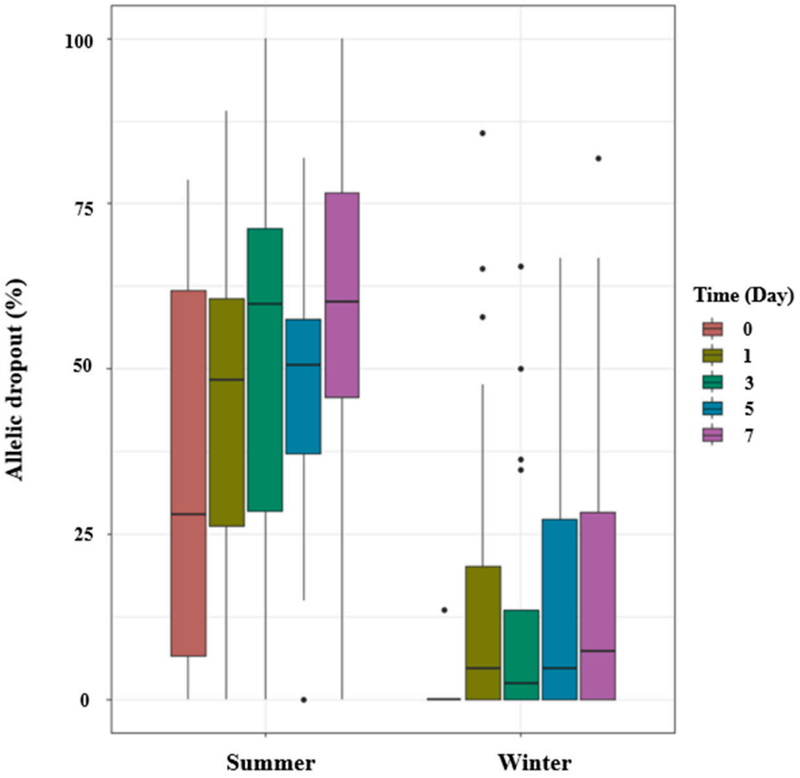
100
75
50
25
0
Allelic dropout (%)
Summer
Winter
Time (Day)
0
1
3
5
7

(c)

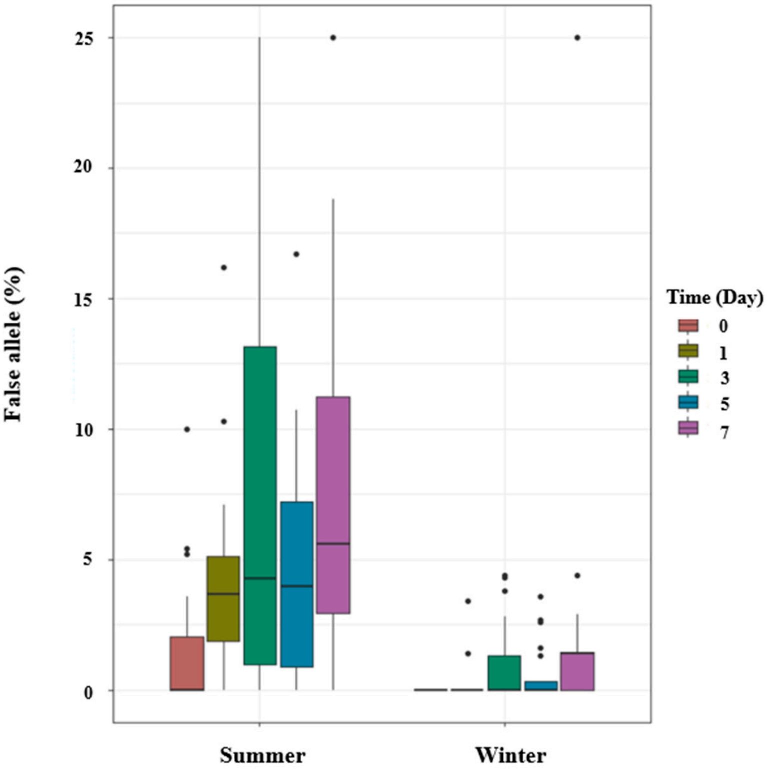

Figure S2. Boxplots showing the distribution of (a) PCR success, (b) allelic dropout, and (c) false allele rates from spraints collected over time in both summer and winter (n = 20 per sample age). The median values are indicated by the lines within each box, and black dots represent outliers in the data.

Table S1. Correlation analysis between response variables (PCR success, allelic dropout, and false allele rates) and weather data (average temperature and relative humidity) in summer and winter. Values presented in the table are Spearman's correlation coefficients (rho), which indicate the strength and direction of the relationship between variables. The values in parentheses are p-values, which are used to determine significance.

Weather variables	Response variables	Sample ages (Day)			
		1	3	5	7
Average temperature (°C)	PCR success	-0.5077 (0.0008***)	-0.4866 (0.0014**)	-0.4515 (0.0034**)	-0.5400 (0.0003***)
	Allelic dropout	0.3870 (0.0136*)	0.4965 (0.0011**)	0.5395 (0.0003***)	0.4887 (0.0014**)
	False allele	0.5828 (7.931e-05***)	0.4624 (0.0027**)	0.5782 (9.32e-05***)	0.4546 (0.0032**)
Average relative humidity (%)	PCR success	-0.4961 (0.0011**)	-0.5467 (0.0003***)	-0.4292 (0.0057**)	-0.5375 (0.0003***)
	Allelic dropout	0.5208 (0.0006***)	0.4972 (0.0011**)	0.4858 (0.0015**)	0.4502 (0.0036**)
	False allele	0.6415 (8.175e-06***)	0.4164 (0.0075**)	0.4172 (0.0074**)	0.4992 (0.0010**)

'*': $p < 0.05$, '**': $p < 0.01$, and '***': $p < 0.001$
